# A novel assay for detection and quantification of C-mannosyl tryptophan in normal or diabetic mice

**DOI:** 10.1038/s41598-019-41278-y

**Published:** 2019-03-18

**Authors:** Sho Sakurai, Yoko Inai, Shiho Minakata, Shino Manabe, Yukishige Ito, Yoshito Ihara

**Affiliations:** 1 Department of Biochemistry, Wakayama Medical University, Wakayama, 641-0012 Japan; 2 RIKEN (The Institute of Physical and Chemical Research), Saitama, 351-0198 Japan

## Abstract

C-Mannosyl tryptophan (C-Man-Trp) is a unique molecule in that an α-mannose is connected to the indole C2 carbon atom of a Trp residue via C-glycosidic linkage. Although serum C-Man-Trp may be a novel biomarker of renal function in humans, the biological significance of C-Man-Trp has yet to be fully investigated. In this study, a novel assay system for C-Man-Trp was established using hydrophilic-interaction liquid chromatography, followed by detecting the fluorescence intensity or mass abundance of C-Man-Trp. Using this system, we systematically assessed the amount of free monomeric C-Man-Trp in different tissues of mice. The tissue level of C-Man-Trp was high, especially in the ovaries and uterus. Other organs with high levels of C-Man-Trp included the brain, spleen, lungs, bladder, and testes. The level was low in skeletal muscle. We also investigated whether the tissue level of C-Man-Trp is affected in diabetes. In KK-Ay diabetic mice, the level of urinary C-Man-Trp excretion was increased, and the tissue levels of C-Man-Trp were decreased in the liver but increased in the kidney. These results demonstrate that C-Man-Trp is differentially distributed in numerous tissues and organs in mice, and the levels are altered by disordered carbohydrate metabolism such as diabetes.

## Introduction

Glycosylation is a major post-translational modification of secretory or membrane proteins. Protein-bound glycans are classified by the nature of their linkage to the protein^1,2^. The glycans are attached to the proteins through N, O, C, or S atoms in the amino acid chains. N-glycan is a major modified form in which N-acetylglucosamine (GlcNAc) is first bound to Asn via an amide bond, the processing of which has been extensively characterized^3^. O-glycan is another major modified form in which mainly N-acetylgalactosamine is bound to the hydroxyl residue of Ser or Thr in proteins^4^. The processing of O-glycan has also been well characterized. In addition to O-glycans, other saccharides, such as mannose, fucose, and GlcNAc, attach directly to Ser or Thr via the hydroxyl residue^1,2^. Complex and diverse glycans in glycoproteins exert a variety of biological functions in the cell^5^.

Monomeric form of C-Mannosyl tryptophan (C-Man-Trp) was isolated from human urine^6,7^ and marine ascidians^8,9^. The C-Man-Trp structure is quite unique in that an α-mannose is connected to the indole C2 carbon atom of a Trp residue via C-glycosidic linkage. It was reported that C-Man-Trp in blood may be a novel biomarker of renal function^10–12^. Furthermore, in other metabolomic studies, upregulation of C-Man-Trp was observed in human plasma and serum in patients with renal dysfunction due to diabetes^13,14^. On the other hand, the structure of C-mannosylated Trp was identified first in human ribonuclease 2 (RNase2)^15^. C-Mannosylation occurs at the first Trp in the consensus amino acid sequence Trp-X-X-Trp in proteins^16^. There are several examples of C-mannosylated proteins included in the thrombospondin type 1 repeat (TSR) superfamily (e.g., thrombospondin, complements, F-spondin, properdin, mindin, etc.), type I cytokine receptor family (e.g., IL-21 receptor, erythropoietin receptor, etc.), and others (e.g., RNase2, MUC5AC, MUC5B, hyaluronidase I, etc.)^17^. C-Mannosylation is carried out by a specific mannosyltransferase located in microsomes, and its responsible gene was identified as DPY-19 in *C*. *elegans*^18^, suggesting that C-mannosylation functions in conventional glycosylation through the secretory pathway. This is consistent with almost all the C-mannosylated proteins found to date being secretory or membrane proteins. Recently, it was reported that DPY19L1 and L3 encode genes for mammalian mannosyltransferases, which possess distinct substrate specificities for the peptide sequence containing the Trp-X-X-Trp motif^19,20^. The functional relevance of glycosylation has been confirmed in numerous cellular events, including cell development, growth, differentiation, and death^5^, but the biological significance of C-mannosylation or C-Man-Trp has yet to be fully investigated. Furthermore, it is currently unknown how and where free monomeric C-Man-Trp is produced in living organisms.

In previous reports, free C-Man-Trp was detected by measuring the specific fluorescence intensity of C-Man-Trp by reversed-phase HPLC^7,11^. However, the assay for C-Man-Trp could be affected by other fluorescent biomolecules similar to C-Man-Trp in biological samples. Here, to accurately detect C-Man-Trp in biological samples, we established a novel assay for C-Man-Trp by measuring the fluorescence intensity and mass abundance of C-Man-Trp under conditions with hydrophilic-interaction liquid chromatography (HILIC). We measured the C-Man-Trp level in tissues and plasma of mice using this system. This is the first report to systematically quantify the level of C-Man-Trp in a variety of tissues and organs of mammals. We also examined the C-Man-Trp levels in different tissues and organs in mice with pathological conditions due to diabetes mellitus to investigate the effects of hyperglycemic conditions on C-Man-Trp in the body.

## Results

### Assay for C-Man-Trp by hydrophilic-interaction liquid chromatography

**Figure 1:**
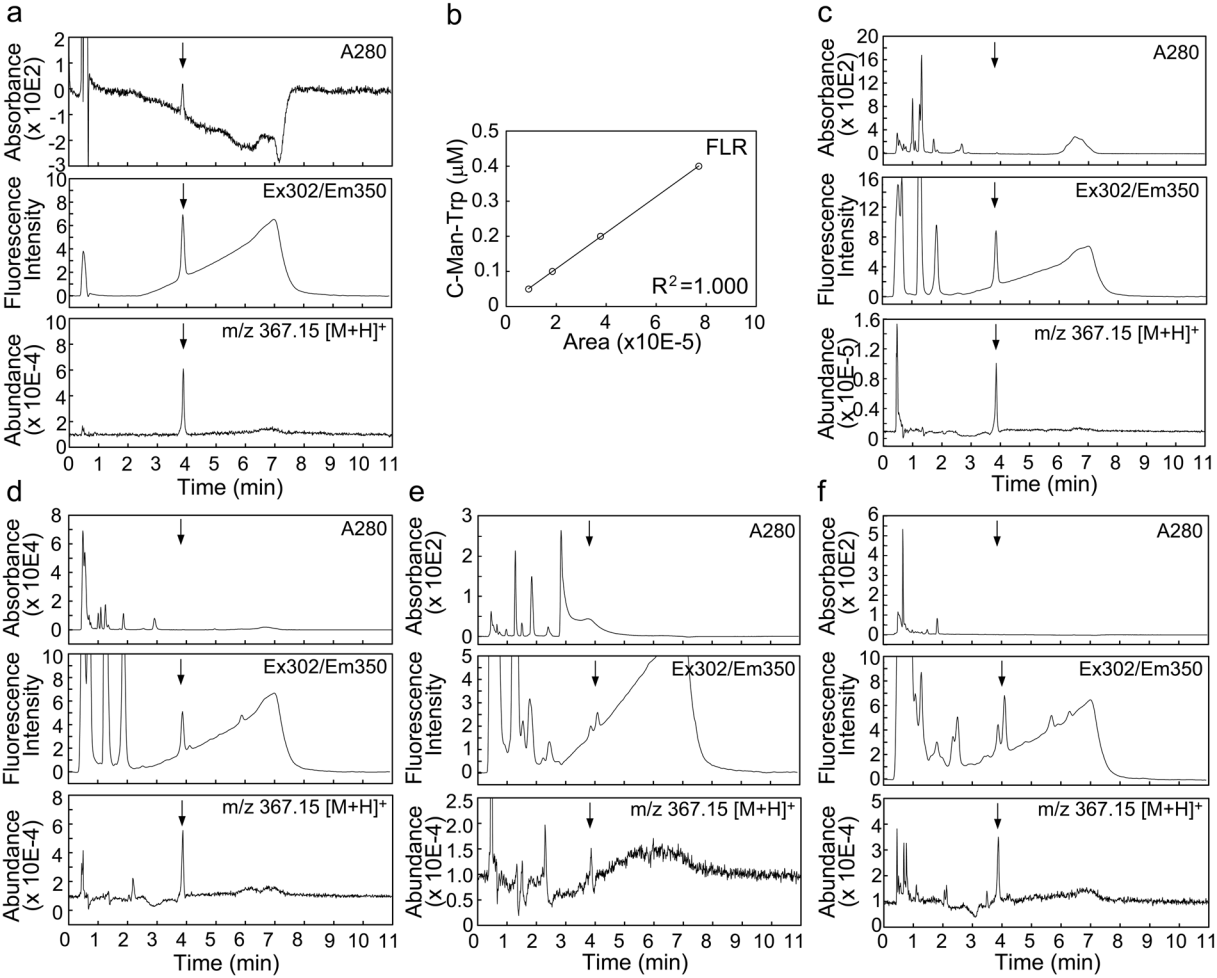
Detection and quantification of C-Man-Trp. Samples were prepared by organic solvent extraction followed by centrifugation, and applied to UPLC as described in Materials and Methods. (**a**) The assay profiles of HILIC to determine C-Man-Trp. Chromatograms of chemically synthesized C-Man-Trp monitored by measuring the absorbance at 280 nm (upper), the fluorescence (excitation at 302 nm/emission at 350 nm) (middle), and the mass abundance at an m/z value of 367.15 [M + H]^+^ (lower). Arrows show the retention time corresponding to C-Man-Trp. (**b**) Calibration curve of C-Man-Trp based on chemically synthesized C-Man-Trp. C-Man-Trp was quantified by measuring the fluorescence intensity (FLR) as described in Materials and Methods. Mouse tissue samples were prepared and applied to UPLC. C-Man-Trp was measured by HILIC in the samples of mouse brain (**c**), liver (**d**), plasma (**e**), and urine (**f**).

In this study, we established a novel assay system for C-Man-Trp using an ultra performance liquid chromatography (UPLC), followed by detecting the fluorescence intensity or mass abundance of C-Man-Trp, as described in Materials and Methods. As shown in Fig. 1a, chemically synthesized C-Man-Trp (arrow) was separated by HILIC, and detected by monitoring the absorbance at 280 nm (upper) and the fluorescence (excitation at 302 nm/emission at 350 nm) (middle). The mass of the target peak considered to be C-Man-Trp was confirmed by mass spectrometry as having an m/z value of 367.15 [M + H]^+^, corresponding to that of C-Man-Trp, and the mass abundance was also measured (lower). The fluorescence (FLR) and mass abundance (QDa) of C-Man-Trp were measured with different concentrations of the standard C-Man-Trp compound. In this study, quantification of C-Man-Trp was carried out for each sample in accordance with the calibration curve shown in Fig. 1b. Typical elution patterns of C-Man-Trp were observed for the brain (Fig. 1c), liver (Fig. 1d), plasma (Fig. 1e), and urine (Fig. 1f). In the brain and liver, C-Man-Trp was detected as a main fluorescence peak with a retention time at 3.9 min. In the plasma and urine, although C-Man-Trp was detected, there were other peaks with retention times close to that of C-Man-Trp.

### Quantification of C-Man-Trp levels in plasma and tissues of C57BL/6 mice

**Figure 2:**
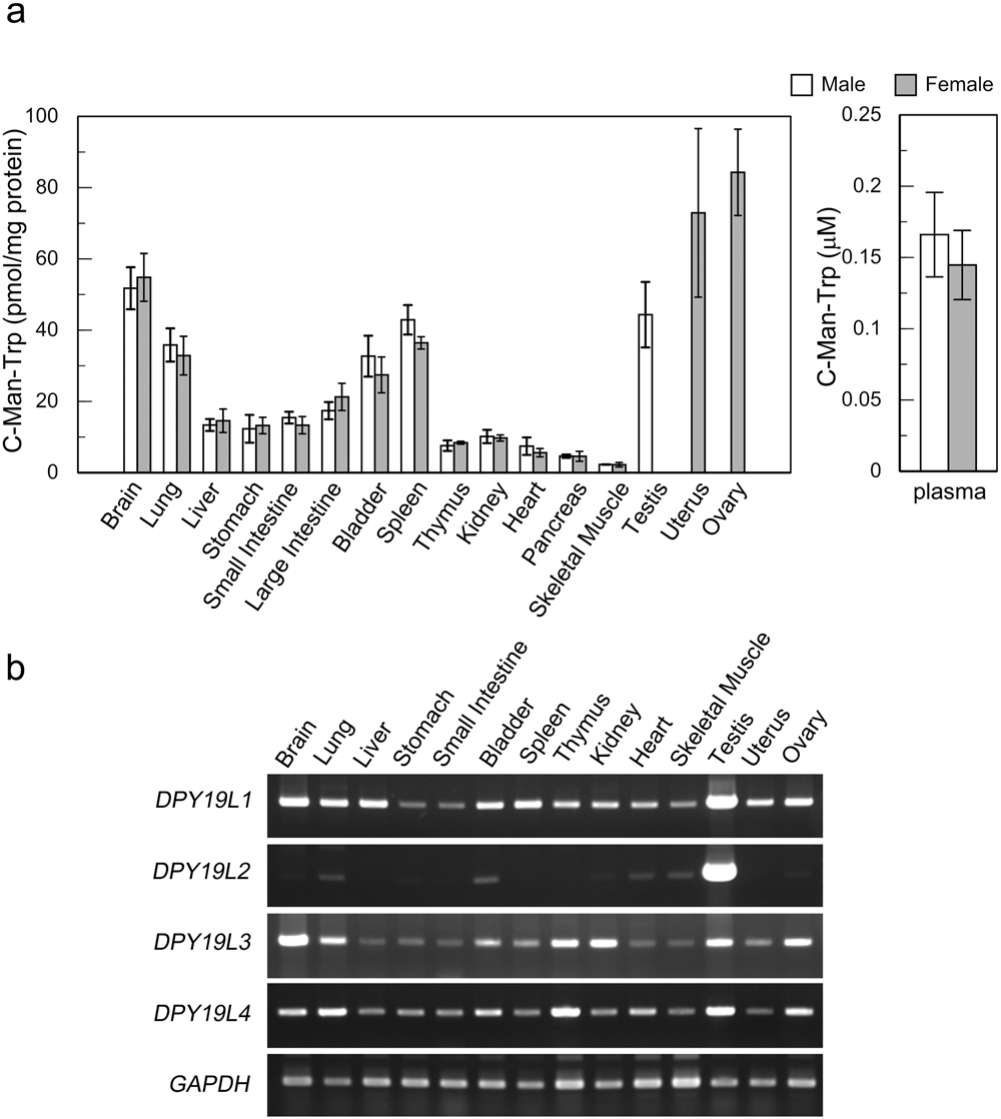
(**a**) C-Man-Trp levels in mouse tissues and plasma. The C-Man-Trp concentration was measured in the samples of tissues from male (n = 6) and female (n = 6) mice (C57BL/6 mice at 6 weeks) as described in Materials and Methods. The levels of C-Man-Trp concentration in Table 1 were graphically illustrated. Data represent the mean ± SD. (**b**) Transcription levels of DPY-19 gene homologues in mouse tissues. The transcripts of DPY19L1-L4 were amplified by RT-PCR and analyzed by agarose gel electrophoresis for the tissues from C57BL/6 mice (6 weeks) as described in Materials and Methods. Cropped gel images are shown and full gel images can be found in Supplementary Fig. S2. Data represent three independent experiments.

Numerous tissue and organ samples were prepared from C57BL/6 mice (male and female, 6 weeks) as described in Materials and Methods. C-Man-Trp levels were examined and quantified for each sample by UPLC-FLR analysis. As shown in Fig. 2a and Table 1, C-Man-Trp was detected in all tested samples, but the levels varied among tissues. The levels of C-Man-Trp were high, especially in the ovaries and uterus. Other organs with high levels of C-Man-Trp included the brain, spleen, lungs, bladder, and testes. The level was low in skeletal muscle. In addition, the levels of C-Man-Trp were similar between the sexes in tested tissues and organs, excluding genitalia. These results suggest that the production or metabolism of C-Man-Trp is differentially regulated in each tissue and organ in mice.

**Table 1:** C-Man-Trp levels in different tissues from C57BL/6 mice at 6 weeks.

Tissue	Male (n = 6)	Female (n = 6)
**Tissue (pmol/mg protein)**		
Brain	51.8 ± 5.9	54.8 ± 6.7
Lung	35.8 ± 4.7	32.9 ± 5.4
Liver	13.4 ± 1.7	14.6 ± 3.3
Stomach	12.3 ± 3.9	13.2 ± 2.3
Small Intestine	15.5 ± 1.7	13.3 ± 2.4
Large Intestine	17.4 ± 2.4	21.3 ± 3.8
Bladder	32.7 ± 5.7	27.5 ± 5.0
Spleen	42.9 ± 4.1	36.4 ± 1.7
Kidney	7.6 ± 1.5	8.4 ± 0.4
Thymus	10.2 ± 1.9	9.7 ± 0.9
Heart	7.4 ± 2.5	5.6 ± 1.2
Pancreas	4.6 ± 0.5	4.6 ± 1.4
Skeletal Muscle	2.3 ± 0.1	2.2 ± 0.6
Testis	44.3 ± 9.2	
Uterus		72.9 ± 23.7
Ovary		84.3 ± 12.1
**Plasma (µM)**		
	0.166 ± 0.030	0.145 ± 0.024

### Transcription levels of DPY-19 gene homologues in selected tissues of C57BL/6 mice

C-Mannosylation was considered to occur at the specific Trp in proteins by a specific C-mannosyltransferase in the cell^16^. DPY19L1 and L3 genes have been experimentally confirmed to encode the mammalian C-mannosyltransferases^19,20^. To investigate the correlation of protein C-mannosylation with the level of C-Man-Trp, the transcription levels of DPY-19 gene homologues (i.e., DPY19L1-L4) were examined in selected tissues of C57BL/6 mice (male and female, 6 weeks) by reverse-transcription polymerase chain reaction (RT-PCR) followed by agarose gel electrophoresis as described in Materials and Methods. As shown in Fig. 2b, the transcript of DPY19L1 was highly expressed in the testes, and moderate expression levels were observed in the brain, lungs, liver, bladder, spleen, thymus, kidney, heart, uterus, and ovaries. The levels were low in the stomach, small intestine, and skeletal muscle. In case of DPY19L2, high level of transcript was expressed especially in the testes, and low expression levels were detected in all of the other tissues. In DPY19L3, the transcript was moderately expressed in the brain, lungs, thymus, kidney, testes, and ovaries. The levels were low in the other tissues. In DPY19L4, the transcript was moderately expressed in the brain, lungs, thymus, testes, and ovaries, and low expression levels were observed in the other tissues. These results were similar to the data available in the Web database, Expression Atlas (EMBL-EBI, http://www.ebi.ac.uk/gxa)^21^.

### Diabetes-related parameters in the plasma and urine of KK-Ay diabetic mice

The KK-Ay mouse is a model of type 2 diabetes with hyperglycemia and albuminuria^22,23^. As shown in Table 2, the data in the present experiments were consistent with those reported in previous studies using the same species^23^. The body weight, blood glucose, urine excretion volume, glycated albumin, and urinary excretion of creatinine in KK-Ay mice (16 weeks) were higher than those in age-matched controls (C57BL/6). In addition, the urine albumin-to-creatinine ratio (ACR) was markedly high in KK-Ay mice (16 weeks), compared with the lower excretion of albumin in age-matched controls. These results are consistent with KK-Ay mice demonstrating characteristics of early stage diabetes with slight renal damage^23^.

**Table 2:** Biochemical parameters in KK-Ay (16 weeks).

	C57BL/6	KK-Ay
Number	5	7
Body Weight (g)	27.00 ± 3.18	46.74 ± 3.65**
Blood Glucose (mg/dl)	174.0 ± 32.1	584.1 ± 145.2**
Urine Volume (ml)	0.78 ± 0.56	5.69 ± 5.28
Glycated Albumin (%)	3.40 ± 0.32	7.84 ± 1.06**
Plasma Creatinine (mg/dl)	0.082 ± 0.022	0.10 ± 0.021
Urine Creatinine Concentration (mg/dl)	45.69 ± 11.68	16.51 ± 7.43**
Urine Creatinine Excretion (mg/day)	0.31 ± 0.16	0.66 ± 0.38
Creatinine Clearance (ml/min/kg)	10.90 ± 7.07	10.89 ± 6.82
Urine Albumin/Creatinine (ACR, µg/mg)	42.1 ± 13.1	2938.4 ± 1105.4**

**P < 0.01 indicates a significant difference versus C57BL/6.

### Plasma level and urinary excretion of C-Man-Trp in KK-Ay mice

**Figure 3:**
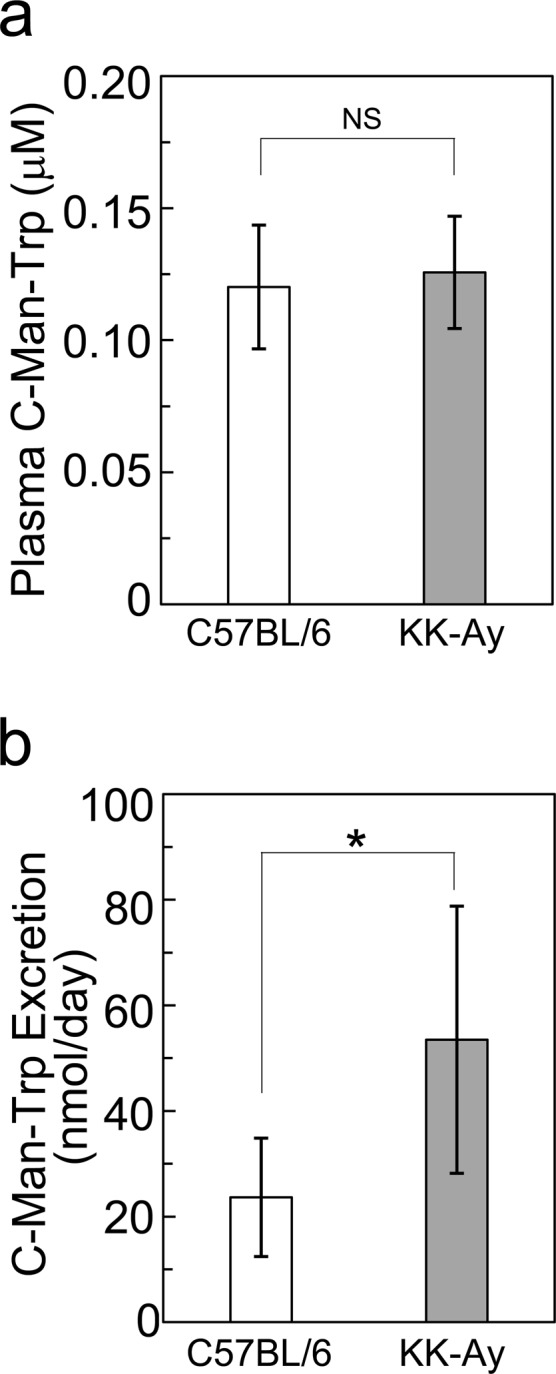
Plasma concentrations and urinary excretion levels of C-Man-Trp in control and KK-Ay diabetic mice. (**a**) The C-Man-Trp concentration in plasma was measured in control (C57BL/6) (n = 5) and KK-Ay (n = 7) mice at 16 weeks as described in Materials and Methods. (**b**) Urinary excretion of C-Man-Trp was measured in C57BL/6 and KK-Ay mice as described in Materials and Methods. Data represent the mean ± SD. *P < 0.05 indicates a significant difference versus C57BL/6 (16 weeks). NS, not significant.

C-Man-Trp levels were examined and quantified for plasma and urine samples as described in Materials and Methods. Plasma creatinine levels were similar between control (C57BL/6) and KK-Ay mice at 16 weeks (Table 2). As shown in Fig. 3a, the plasma C-Man-Trp levels were comparable between control and KK-Ay mice.

The level of urinary creatinine excretion was increased in KK-Ay mice by more than two-fold compared with that in age-matched controls (Table 2). As shown in Fig. 3b, the level of urinary C-Man-Trp excretion was also significantly increased in KK-Ay mice compared with that in age-matched controls. Together, these results suggest that the urinary excretion of both creatinine and C-Man-Trp increases in diabetes.

### Renal clearance of C-Man-Trp in KK-Ay mice

**Figure 4:**
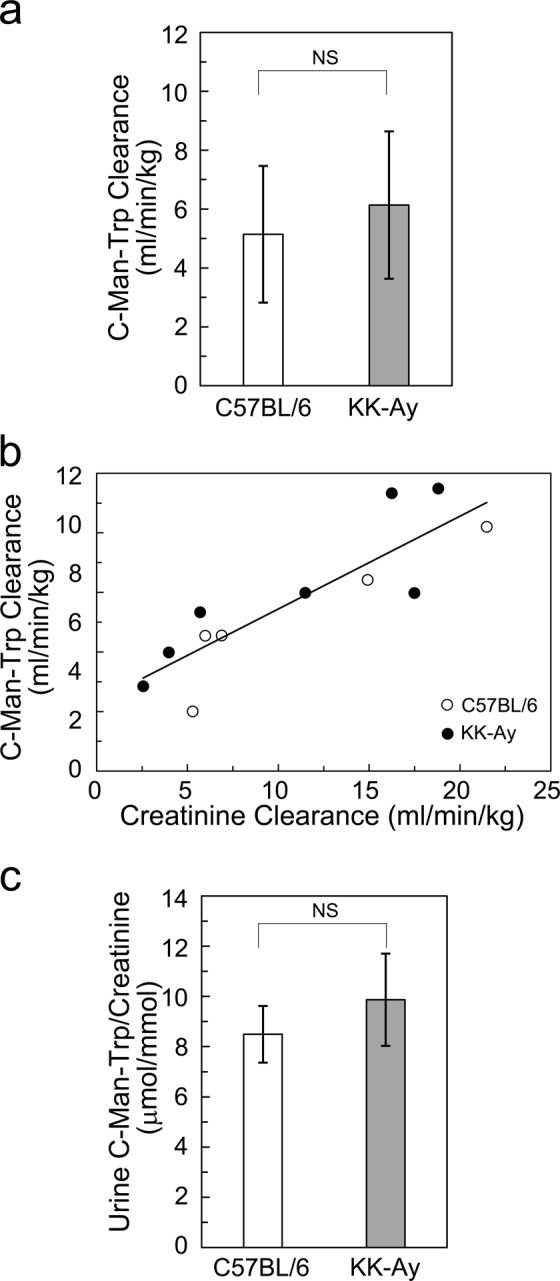
C-Man-Trp clearance and urine C-Man-Trp/creatinine in control and KK-Ay diabetic mice. (**a**) C-Man-Trp clearance was measured in control (C57BL/6) (n = 5) and KK-Ay (n = 7) mice at 16 weeks as described in Materials and Methods. (**b**) The correlation between creatinine clearance and C-Man-Trp clearance was analyzed by Pearson’s correlation test in C57BL/6 and KK-Ay mice. (**c**) Urinary C-Man-Trp/creatinine was measured in C57BL/6 and KK-Ay mice as described in Materials and Methods. Data represent the mean ± SD. NS, not significant.

The creatinine clearance levels were similar between control (C57BL/6) and KK-Ay mice at 16 weeks (Table 2). As shown in Fig. 4a, C-Man-Trp clearance levels were comparable between control and KK-Ay mice. Furthermore, the C-Man-Trp clearance was positively correlated with the creatinine clearance (r = 0.866, *P* < 0.01) in control and KK-Ay mice at 16 weeks (Fig. 4b). Next, we estimated the urinary concentration ratio of C-Man-Trp to creatinine (C-Man-Trp/creatinine, μmol/mmol) in control and KK-Ay mice. As shown in Fig. 4c, the ratio did not differ between control and KK-Ay mice at 16 weeks. Therefore, the renal clearance of C-Man-Trp was less affected in KK-Ay mice.

### The cellular level of C-Man-Trp in selected organs of KK-Ay mice

**Figure 5:**
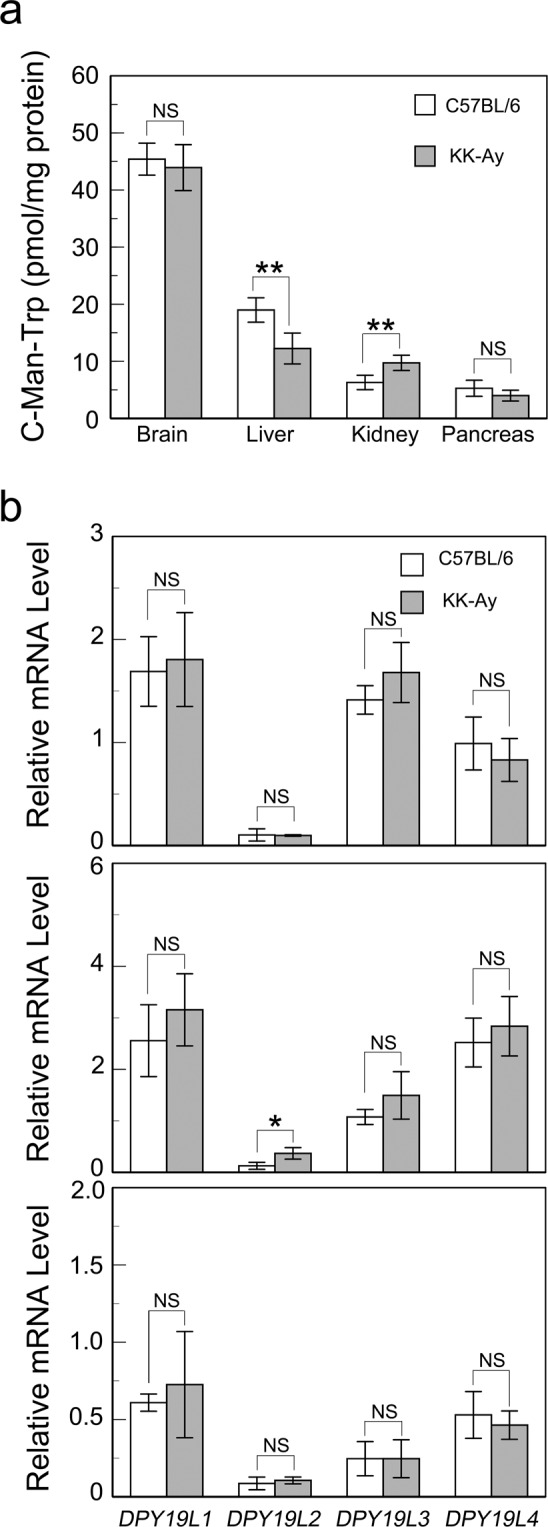
(**a**) The levels of C-Man-Trp in selected tissues of diabetic model mice. The C-Man-Trp concentration was measured in the samples of tissues from control (C57BL/6) (n = 5) and KK-Ay (n = 7) mice at 16 weeks as described in Materials and Methods. Data represent the mean ± SD. **P < 0.01 indicates a significant difference versus C57BL/6. NS, not significant. (**b**) Transcription levels of DPY-19 gene homologues in brain (upper), liver (middle), and kidney (lower), of diabetic model mice. The transcripts of DPY19L1-L4 were amplified by RT-PCR and analyzed by agarose gel electrophoresis for the tissues from control (C57BL/6 mice, 16 weeks) and KK-Ay (16 weeks) mice as described in Materials and Methods. Band intensity was quantified as described in Materials and Methods. Data represent the mean ± SD. *P < 0.05 indicates a significant difference versus C57BL/6. NS, not significant.

In KK-Ay mice at 16 weeks, the C-Man-Trp level was higher in urinary secretion than that in age-matched controls (Fig. 3b). To investigate whether the amount of C-Man-Trp is altered in specific organs of diabetic mice, the level of C-Man-Trp was examined in selected tissues, such as the brain, liver, kidney, and pancreas, of KK-Ay mice. As shown in Fig. 5a, in KK-Ay mice at 16 weeks, the levels of C-Man-Trp were decreased in the liver but increased in the kidney compared with those in age-matched controls (C57BL/6). In the brain and pancreas, the levels were comparable between control and KK-Ay mice. To further investigate whether protein C-mannosylation is affected in the tissues of diabetic mice, the transcription levels of DPY-19 gene homologues (i.e., DPY19L1-L4) were examined in the brain, liver, and kidney, and compared between control and KK-Ay mice at 16 weeks. As shown in Fig. 5b, there were no significant differences in the levels of DPY19L1 or L3 in all of the tested tissues between control and KK-Ay mice, indicating that the transcription levels of DPY19L1 and L3 are not affected in the tissues of diabetic mice. Although the basal level of DPY19L2 was quite low in the liver of control mice (Fig. 2b), it was significantly increased in the liver of KK-Ay mice compared with that of the control mice by unknown reason (Fig. 5b, middle). Collectively, these results also indicated that the expressions of C-mannosyltransferase genes (i.e., DPY19L1 and L3) are not directly correlated with the changes in the level of C-Man-Trp especially in the liver and kidney of diabetic KK-Ay mice.

## Discussion

Monomeric C-Man-Trp was detected and quantified in human urine and plasma by measuring the specific fluorescence intensity of C-Man-Trp by reversed-phase HPLC^7,11^. In these studies, C-Man-Trp was separated from other endogenous compounds in the urine or plasma, and identified by solely tracking its specific fluorescence. However, it was unclear whether other fluorescent biomolecules similar to C-Man-Trp affected the measurement of C-Man-Trp in the samples. To more accurately detect C-Man-Trp in biological samples, we established a novel assay for C-Man-Trp by measuring the fluorescence intensity and mass abundance of C-Man-Trp under conditions with HILIC as described in Materials and Methods. We also observed tissue extract samples that had fluorescent contaminants with a similar retention time as C-Man-Trp in the chromatography profile. In mouse urine (Fig. 1f), as the fluorescent peak of C-Man-Trp partly overlaps with another peak in the chromatography profile, the peak molecule was further confirmed as C-Man-Trp by measuring the mass abundance by mass spectrometry. Indeed, to detect and measure C-Man-Trp in biological samples, the present assay system is likely more reliable than previous methods.

In previous reports, free C-Man-Trp was detected in urine and plasma of humans^6,7^ and in extracts of marine ascidians^8,9^. However, the tissue or organ distribution of C-Man-Trp has not been well studied in mammals. In this study, we examined the distribution of C-Man-Trp in several tissues and organs from mice. As shown in Fig. 2a and Table 1, the level of C-Man-Trp is high, especially in the ovaries and uterus. The level is also high in the brain, spleen, lungs, bladder, and testes, but is low in skeletal muscle. These results revealed that C-Man-Trp is distributed throughout numerous tissues and organs in mammals. C-Mannosylation is believed to occur enzymatically on the first Trp in the consensus amino acid sequence of Trp-X-X-Trp/Phe/Cys of secretory or membrane proteins in microsomes^16,19,20^. The responsible mammalian genes for C-mannosyltransferase were recently identified as DPY-19 gene homologues such as DPY19 L1 and L3^19,20^. Monomeric C-Man-Trp is suspected to be a catabolic product of C-mannosylated proteins in different tissues or organs because it is detected in extracellular fractions, such as plasma and urine, in mammals. To investigate how the level of C-Man-Trp is differentially controlled in the tissues or organs of mice, we examined the transcriptional expressions of DPY-19 gene homologues in the tissues of C57BL/6 mice (Fig. 2b). High or moderate levels of DPY19L1 expression seem to be compatible with the levels of C-Man-Trp in the brain, spleen, lungs, bladder, testes, uterus, and ovaries. Low levels of DPY19L1 expression also seem to be compatible with the low levels of C-Man-Trp in the stomach, small intestine, and skeletal muscle. However, in the liver, the level of DPY19L1 expression is moderate in spite of the low level of C-Man-Trp. On the other hand, DPY19L3 is moderately expressed in the brain, lungs, testes, and ovaries, and it seems consistent with the levels of C-Man-Trp in the corresponding tissues or organs. Low levels of DPY19L3 expression also seem to be compatible with the low levels of C-Man-Trp in the liver, stomach, small intestine, heart, and skeletal muscle. In contrast, in the thymus and kidney, the moderate expression levels of DPY19L3 seem inconsistent with the low levels of C-Man-Trp. Although it has not been shown whether DPY19L2 and/or DPY19L4 could exert C-mannosyltransferase activity^19,20^, high level of DPY19L2 is expressed especially in the testes, and DPY19L4 is moderately expressed in almost tissues or organs of mice (Fig. 2b). Taken together, these results indicated that C-mannosyltransferase genes are differentially expressed in various tissues or organs of mice, and the transcription levels of either DPY19L1 or L3 are reflected in the levels of C-Man-Trp in the tissues. Thus, the tissue level of free C-Man-Trp is presumably affected in part by the biosynthesis of protein C-mannosylation through the specific expression of C-mannosyltransferase genes, such as DPY19L1 and/or L3. Further investigation is still required to clarify how the level of C-Man-Trp is differentially regulated in each tissue or organ in mammals.

In regard to biological significance of C-Man-Trp in blood, there have been various reports that plasma or serum C-Man-Trp increases in renal dysfunction in humans, proposing free C-Man-Trp as a biomarker in blood to assess renal function^10–14,24^. Yonemura *et al*. reported that the concentration of C-Man-Trp is a more reliable diagnostic parameter than that of serum creatinine because the C-Man-Trp level is not affected by age or muscle mass^12^, although C-Man-Trp was identified in fasting blood as a specific metabolite, highly correlated with age and aging traits such as lung function and bone mineral density^25^. Niewczas *et al*. reported that the serum C-Man-Trp significantly increases with progression of renal damage in type 2 diabetes patients, suggesting C-Man-Trp as a serum marker to assess the progression from early-stage to end-stage renal disease in type 2 diabetes patients^13^. However, in that study, the level of serum C-Man-Trp was especially higher in the progressing patients than the non-progressing patients in diabetes, suggesting that serum C-Man-Trp is upregulated simply in case of progressed renal damage irrelevant to hyperglycemia. In other metabolomics studies, the serum levels of C-Man-Trp and pseudouridine were increased in renal dysfunction with a low prevalence of diabetes in humans^10,24^. In addition, combined assessment of C-Man-Trp with other serum metabolites (e.g., pseudouridine and N-acetylthreonine) could be more effective to assess the renal dysfunction in humans^10,14^. Collectively, these studies demonstrated that serum C-Man-Trp is a possible biomarker for progressed renal damage. In the present study, we investigated the plasma/urine C-Man-Trp levels and albumin excretion in urine in type 2 diabetic model mice with slight renal damage. In KK-Ay mice at 16 weeks, diabetes-related albuminuria (i.e., ACR) was increased (Table 2), and the level of C-Man-Trp was not changed in the plasma, compared with those in age-matched controls (Fig. 3a). As the KK-Ay mouse is a model of early-stage type 2 diabetes^23^, our observations of less increase in serum C-Man-Trp in KK-Ay mice may be compatible with the pathological state of the early stage of diabetic nephropathy without renal dysfunction. However, it is not known whether serum C-Man-Trp is a useful marker to assess the renal dysfunction in the early stage of renal abnormality with microalbuminuria. Further investigation is required to establish the usefulness of serum C-Man-Trp for the precise assessment of renal functions.

Meanwhile, the level of C-Man-Trp was increased in urinary excretion in KK-Ay mice (Fig. 3b). This suggests that the increased urinary excretion of C-Man-Trp may represent some of the altered renal functions in the type 2 diabetes mice. On the other hand, we also produced the streptozotocin (STZ)-induced diabetic mouse, a model of type 1 diabetes with hyperglycemia^26^, and examined the mice for the C-Man-Trp assay. Albuminuria was not observed in STZ mice, and it was consistent with STZ mice demonstrating characteristics of early stage diabetes without renal damage (Supplementary Table S1). In terms of plasma C-Man-Trp concentration, urinary excretion of C-Man-Trp, and C-Man-Trp clearance, there were no significant differences between STZ and control mice (Supplementary Fig. S1). The results suggest that, in the absence of albuminuria, urinary excretion of C-Man-Trp is not affected even under the hyperglycemic conditions in STZ mice. Furthermore, urine C-Man-Trp/creatinine ratio was significantly decreased in STZ mice compared with that in the control mice by unknown reason (Supplementary Fig. S1). Collectively, these results suggest that the level of urinary C-Man-Trp excretion may be a key factor to assess the early renal abnormality with microalbuminuria irrelevant to hyperglycemia. Therefore, further studies are required to clarify how the level of plasma/urine C-Man-Trp is related to the progression in the early stage of renal dysfunction in diabetes or other diseases.

The levels of C-Man-Trp were significantly decreased, especially in the liver, in both KK-Ay and STZ mice compared with those in the control mice (Fig. 5a and Supplementary Fig. S1), suggesting that the tissue levels of C-Man-Trp are affected under the hyperglycemic conditions of diabetes. However, as shown in Fig. 5b, there were no changes in the transcription levels of DPY19L1 and L3 in the liver and kidney between control and KK-Ay mice, suggesting that anabolic protein C-mannosylation is not much affected under hyperglycemia. In the liver and kidney of KK-Ay mice, the level of C-Man-Trp may be controlled not only by the anabolic C-mannosylation of proteins but also by the catabolic degradation of C-mannosylated proteins and the cellular transport of C-Man-Trp. Thus, other regulatory pathways related to the catabolism or the transport for C-Man-Trp is presumably involved in the changes in the tissue level of C-Man-Trp in KK-Ay mice. Further investigation is required to clarify how the level of C-Man-Trp is regulated in each tissue or organ in diabetic mice. Moreover, it is of interest to know whether free C-Man-Trp exerts specific biological functions in each tissue or organ.

In this study, we systematically assessed the amount of free monomeric C-Man-Trp in different tissues, organs, plasma, and urine of mice. Free C-Man-Trp levels vary among tissues and organs. We also found that the expression levels of C-mannosyltransferase genes (i.e., DPY19L1 and L3) are partially reflected in the levels of C-Man-Trp in each tissue, suggesting that the biosynthesis of C-mannosylated proteins is correlated with the tissue level of C-Man-Trp to some extent. In diabetes mellitus, a disease causing impaired glucose homeostasis, protein glycosylation and glycan metabolism are influenced by pathological processes^27,28^. In diabetic KK-Ay mice, the level of C-Man-Trp was increased in urinary excretion, and the tissue levels were decreased in the liver but increased in the kidney, suggesting that the metabolism of free C-Man-Trp is also greatly influenced in diabetes. C-Man-Trp is still enigmatic regarding its biosynthesis, catabolism, and functions. Thus, by using this novel HILIC assay to determine free C-Man-Trp, further investigation is required to clarify the biological significance of C-Man-Trp.

## Materials and Methods

### Materials

Reagents used in the study were of high quality and from Sigma (St. Louis, MO), Waters (Milford, MA), or Wako Pure Chemical Industries, Ltd. (Osaka, Japan).

### Synthesis of C2-α-C-mannosyl-L-tryptophan

C2-α-C-mannosyl-L-tryptophan (C-Man-Trp) was synthesized as previously described^29^. The purity and identity of the final product were verified by ^1^H NMR spectroscopy and matrix-assisted laser desorption ionization (MALDI) mass spectrometry. The proton chemical shifts and coupling constants are consistent with those reported previously. The mass on MALDI mass spectrometry was consistent with the expected mass of the correct product.

### Experimental mice

C57BL/6 mice were purchased from Kiwa Laboratory Animals Co., Ltd., Japan, unless otherwise stated. Male type 2 diabetic KK-Ay mice were obtained from CLEA Japan. All mice were housed in autoclaved cages, and maintained with food and water ad libitum. All experimental procedures were approved by the Wakayama Medical University Animal Care and Use Committee, and conducted in accordance with the regulations for animal experiments at Wakayama Medical University.

### Biochemical and metabolic parameters

Blood glucose levels were measured using a glucose measurement device (GLUCOCARD GT-1641, Arkray, Kyoto, Japan). Other biochemical parameters in plasma and urine were analyzed by Nagahama Life Science Laboratory (Nagahama, Japan). Creatinine levels in plasma or urine were measured using corresponding enzyme kits (Wako Pure Chemical). Plasma glycated albumin levels were measured by the enzymatic method (LusicaGA-L, Sekisui Medical Co., Ltd., Tokyo, Japan). Urinary albumin levels were determined by the enzyme-linked immunosorbent assay kit AKRAL-121 (Shibayagi, Gunma, Japan).

### Sample preparation

On the day before sacrifice, mice were transferred to metabolic cages for 24 h for urinalysis. Mice were anesthetized with isofulrane and blood was sampled from the heart. After cervical spine fracture dislocation, tissues were excised, rinsed with ice-cold phosphate-buffered saline (PBS, pH 7.2), and cut into small pieces. The samples were frozen in liquid nitrogen and stored at −80 °C until use. For measurement of C-Man-Trp, the frozen samples were homogenized in extraction solution (methanol: acetonitrile: formic acid = 80: 19.9: 0.1), and centrifuged at 12,000 × g for 15 min at 4 °C, and the supernatants were collected. The liquid samples (urine or plasma) were diluted in extraction solution, and the supernatants were similarly collected. All supernatants were further filtered using a 0.45-μm PVDF syringe filter before liquid chromatography analysis.

### UPLC conditions

The samples for the C-Man-Trp assay were subjected to the Waters Acquity UPLC (ultra performance liquid chromatography) H-Class system (Waters Corp., Milford, MA) equipped with a photodiode array detector, fluorescence detector, isocratic solvent manager, and quadrupole single mass spectrometer with an electrospray ionization (ESI) source (Waters H-Class QDa system). Separation was carried out on an Aquity UPLC BEH Amide column (2.1 × 100 mm, 1.7 μm) maintained at 40 °C. Both solvent A (water: acetonitrile = 15: 85) and solvent B (water: acetonitrile = 45: 55) contained 0.025% formic acid. C-Man-Trp was eluted in a linear gradient at a flow rate of 0.5 ml/min with 100% solvent A for 1 min, 0–100% solvent B for 5 min, and the column was equilibrated with solvent A for 5 min before the next injection. For validation of the C-Man-Trp peak, the eluate was divided into two flow passes with an isocratic solvent manger. One of the passes was connected into a photodiode array detector followed by a fluorescence detector, and the other was connected to a quadrupole single mass spectrometer. Fluorescence detection (excitation at 302 nm/emission at 350 nm), and absorbance detection at 280 nm were used to monitor C-Man-Trp. Mass detection of the compound was performed in the positive ionization mode: nebulizing gas (N_2_) flow, 20 L/min; probe temperature, 600 °C; cone voltage, 15 V, selected ion recording mode at an m/z value of 367.15 [M + H]^+^ for C-Man-Trp. Empower 3 software was used to collect and process data.

C-Man-Trp was chemically synthesized as described, and used as a standard compound in the assays. The detection limits of C-Man-Trp based on the measured fluorescence and mass abundance were 10 nM and 25 nM, respectively. Extraction recovery rates were evaluated by comparing the analytes from tissues (brain and liver), plasma, and urine with those of the analytes including a known amount of C-Man-Trp. The extraction recovery rates of C-Man-Trp detected by fluorescence were between 90.9% (plasma) and 100.2% (brain). Mass detection gave extraction recovery rates between 100.7% (brain) and 101.3% (liver), although it was not applicable for the plasma samples because of the low concentration of C-Man-Trp.

### Reverse-transcription polymerase chain reaction (RT-PCR)

Total RNA was obtained from mouse tissue samples using TRIzol reagent (Thermo Fisher Scientific, Rockford, IL) according to the manufacturer’s instruction. RNA was reverse transcribed and amplified using a Prime Script One Step RT-PCR Kit Ver.2 (Takara Bio Inc., Shiga, Japan). Basal condition of the PCR was run for 26 cycles of 94 °C for 30 sec /56 °C for 30 sec /72 °C for 80 sec for DPY19L1 and L2. For DPY19L3, the annealing temperature was changed to 63 °C in the basal condition above. For DPY19L4, the extension time was changed to 60 sec in the basal condition. For GAPDH, the PCR was run for 25 cycles of 94 °C for 30 sec /56 °C for 30 sec /72 °C for 60 sec. Primer sequences were as follows: for mouse DPY19L1 (NCBI accession number NM_172920), forward: 5′-TGCATCATTATTTGCCGTGT-3′; reverse: 5′-CAGTCACTGTCTGAGAACTT-3′, for mouse DPY19L2 (NCBI accession number NM_001166207), forward: 5′-ACAAAACCTGGTTGCAGCAT-3′; reverse: 5′-CCTCTTTGCTCCACACAGAA-3′, for mouse DPY19L3 (NCBI accession number NM_178704), forward: 5′-ATGGCTTTCTCACCAGTGCT-3′; reverse: 5′-GTGGCTCCGTGTATCAGGTT-3′, for mouse DPY19L4 (NCBI accession number NM_001081201), forward: 5′-CCCGAACTTTGGATGACACT-3′; reverse: 5′-CGCTACAGGGAAGCTTTACG-3′, and for mouse GAPDH (NCBI accession number NM_001289726), forward: 5′-GGATTTGGCCGTATTGGGCG-3′; reverse: 5′-CAGTAGAGGCAGGGATGATG-3′. The PCR products were electrophoresed using agarose gel (1%), and the bands were visualized by staining with ethidium bromide. The results were quantified densitometrically using CS Analyzer, version 3.00 (ATTO Corp., Tokyo, Japan). GAPDH, a house keeping gene, was used for normalization. Deduced molecular sizes of the PCR products were 1234 bp, 1012 bp, 934 bp, 906 bp, and 607 bp, for DPY19L1, DPY19L2, DPY19L3, DPY19L4, and GAPDH, respectively.

### Statistical analysis

Data are presented as means ± SD. Statistical analysis was performed using the unpaired Student’s t-test (GraphPad Prism7; GraphPad software). *P* < 0.05 was considered significant.

## Supplementary information

Supplementary Information
