# Supplementary material for: A novel assay for detection and quantification of C-mannosyl tryptophan in normal or diabetic mice
Source: Sci Rep. 2019 Mar 18;9:4675. doi: 10.1038/s41598-019-41278-y (PMC6423288; doi:10.1038/s41598-019-41278-y)
Supplement: Supplementary file 1 — Supplementary Information [file 41598_2019_41278_MOESM1_ESM.docx]

**Supplementary Information**

A novel assay for detection and quantification of C-mannosyl tryptophan in normal or diabetic mice

Sho Sakurai^1,2^, Yoko Inai^1,2^, Shiho Minakata^1^, Shino Manabe^3^, Yukishige Ito^3^, and Yoshito Ihara^1,*^

^1^Department of Biochemistry, Wakayama Medical University, Wakayama 641-0012, Japan and ^3^RIKEN (The Institute of Physical and Chemical Research), Saitama 351-0198, Japan

^2^These authors equally contributed to this work.

^*^Address correspondence to: Yoshito Ihara

Department of Biochemistry, School of Medicine, Wakayama Medical University

811-1 Kimiidera, Wakayama 641-0012, Japan

Phone/Fax: 81-73-441-0628, E-mail: y-ihara@wakayama-med.ac.jp

**Supplementary Methods**

*The streptozotocin (STZ)-induced diabetic mice*

Male C57BL/6 mice (6 weeks old) were given a single intraperitoneal injection at 200 mg STZ/kg body weight to induce experimental diabetes. STZ was dissolved in 50 mM sodium citrate buffer (pH 4.5) just prior to injection, and used within 15 min. Three days later, all mice exhibited hyperglycemia (random blood glucose levels were higher than 300 mg/dl). In STZ mice at 8 weeks, the blood glucose level and urine excretion volume were significantly increased, and the body weight was significantly decreased compared with those of age-matched controls. Urinary excretion of creatinine was likely increased in STZ mice, although the level in plasma was not. Albuminuria was not observed in STZ mice. These results are consistent with STZ mice demonstrating characteristics of early stage diabetes without renal damage.

**
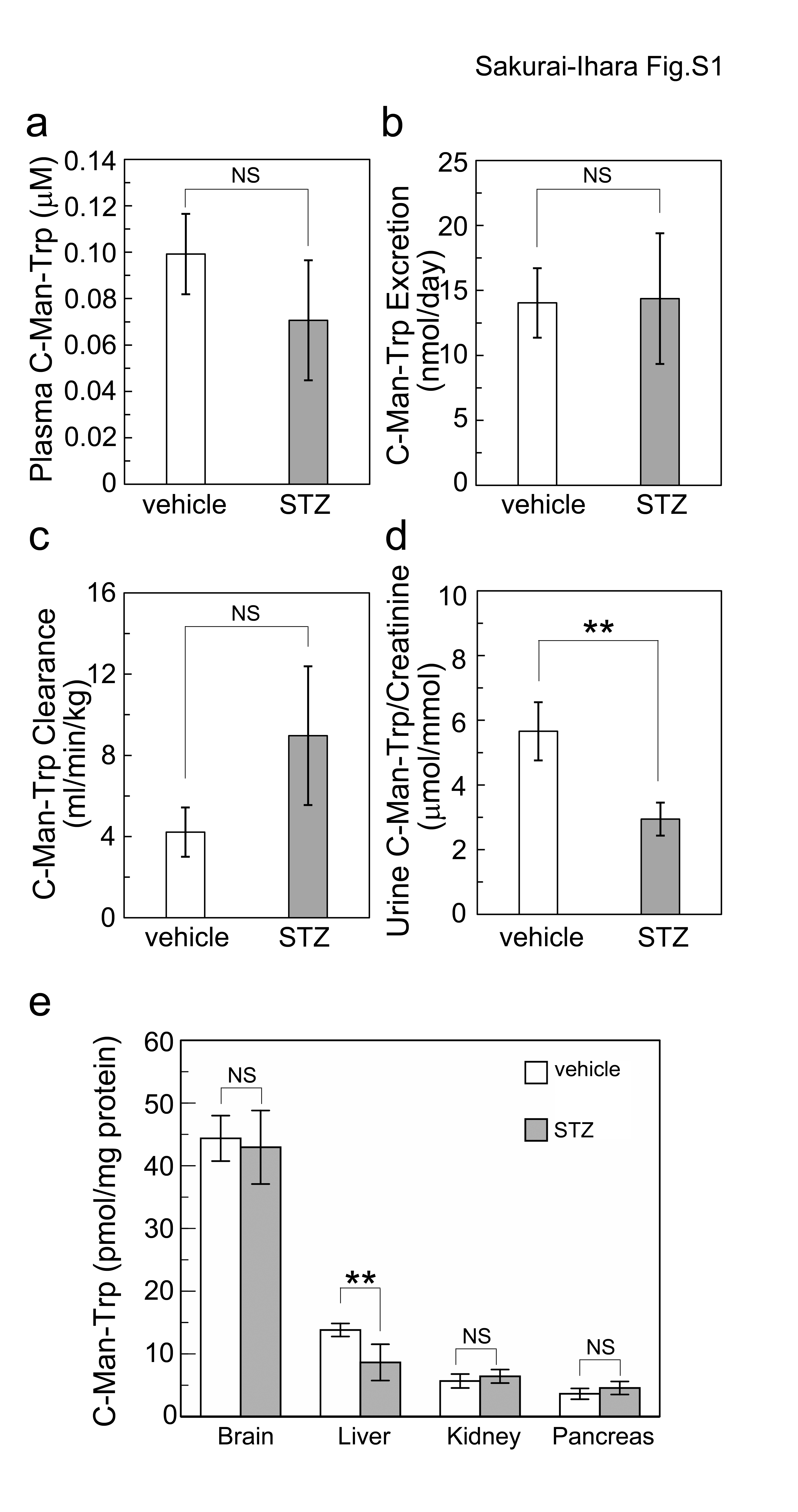
**

**Supplementary Fig. S1.**

Plasma concentrations and urinary excretion levels of C-Man-Trp, C-Man-Trp clearance, and urine C-Man-Trp/creatinine in control and STZ-induced diabetic mice. (a) The C-Man-Trp concentration in plasma was measured in control (vehicle) (n = 6) and STZ (n = 5) mice at 8 weeks. (b) Urinary excretion of C-Man-Trp was measured in vehicle and STZ mice. (c) C-Man-Trp clearance was measured in vehicle and STZ mice. (d) Urinary C-Man-Trp/creatinine was measured in vehicle and STZ mice. (e) The levels of C-Man-Trp in selected tissues of diabetic model mice. Data represent the mean ± SD. ***P* < 0.01 indicates a significant difference versus vehicle. NS, not significant.

**
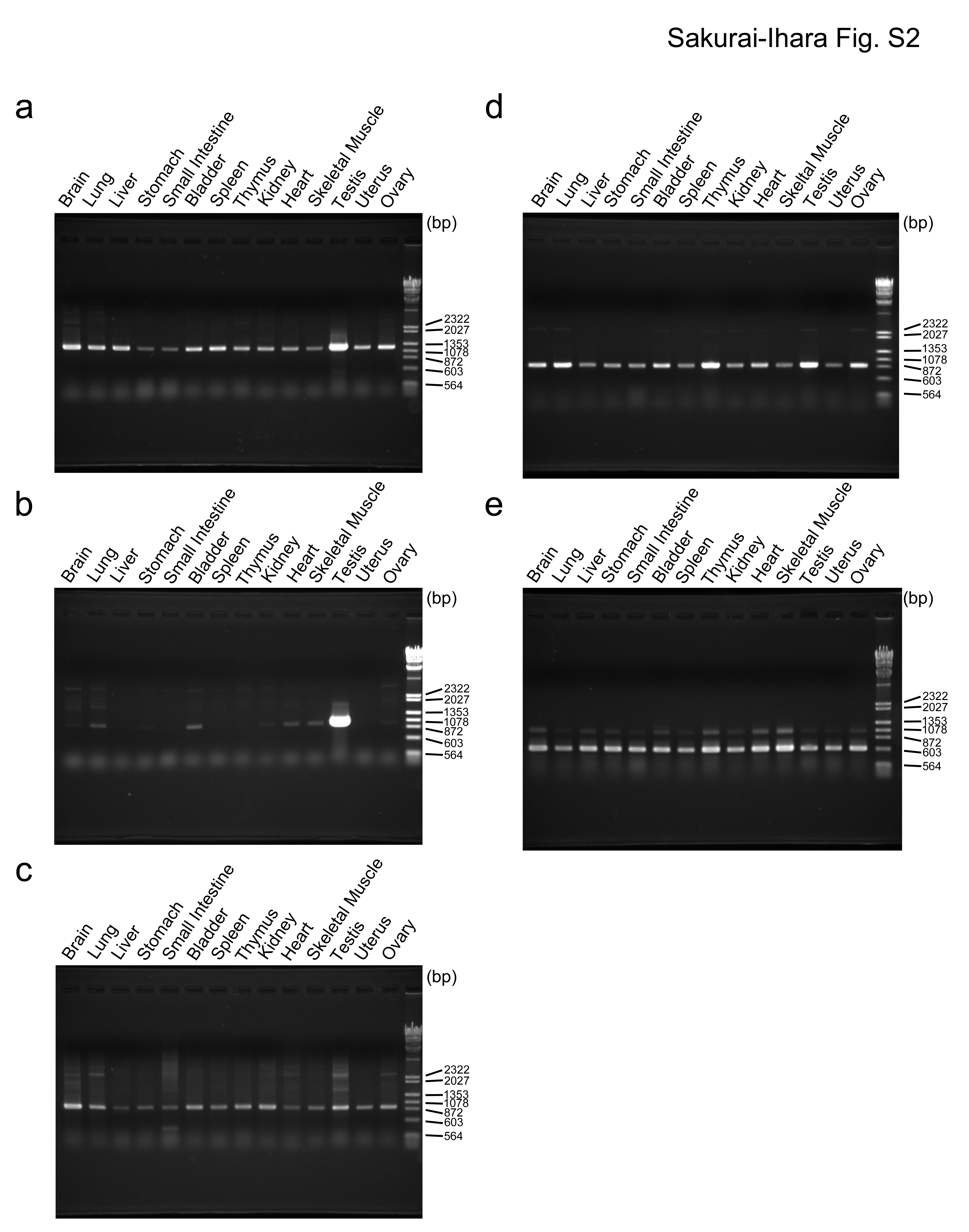
**

**Supplementary Fig. S2.**

Raw image data of agarose gels stained with ethidium bromide for Fig. 2b. The transcripts of DPY19L1-L4 and GAPDH were amplified by RT-PCR and analyzed by agarose gel electrophoresis for the tissues from C57BL/6 mice (6 weeks) as described in Materials and Methods. The transcripts are DPY19L1 (a), DPY19L2 (b), DPY19L3 (c), DPY19L4 (d), and GAPDH (e).

**Supplementary Table S1.** Biochemical parameters in STZ-induced diabetic mice (8 weeks).

|  | Vehicle | STZ |
| --- | --- | --- |
|  | 8w | 8w |
| Number | 6 | 5 |
| Body Weight (g) | 23.98 ± 0.84 | 17.06 ±1.55** |
| Blood Glucose (mg/dl) | 136.7 ± 17.1 | 744.6 ± 142.6** |
| Urine Volume (ml) | 0.51 ± 0.22 | 29.53 ± 7.16** |
| Plasma Creatinine (mg/dl) | 0.095 ± 0.018 | 0.10 ± 0.013 |
| Urine Creatinine Concentration (mg/dl) | 60.34 ± 11.09 | 1.85 ± 0.06** |
| Urine Creatinine Excretion (mg/day) | 0.29 ± 0.10 | 0.55 ± 0.12* |
| Creatinine Clearance (ml/min/kg) | 8.83 ± 1.98 | 21.4 ± 3.68** |

***P* < 0.01, **P* < 0.05 versus vehicle.
